# AI-driven educational transformation in ICT: Improving adaptability, sentiment, and academic performance with advanced machine learning

**DOI:** 10.1371/journal.pone.0317519

**Published:** 2025-05-19

**Authors:** Azhar Imran, Jianqiang Li, Ahmad Alshammari

**Affiliations:** 1 School of Software Engineering, Beijing University of Technology, Beijing, China; 2 Department of Creative Technologies, Air University, Islamabad, Pakistan; 3 Department of Computer Sciences, Faculty of Computing and Information Technology, Northern Border University, Rafha, Kingdom of Saudi Arabia; BRAC Business School, BRAC University, BANGLADESH

## Abstract

This study significantly contributes to the sphere of educational technology by deploying state-of-the-art machine learning and deep learning strategies for meaningful changes in education. The hybrid stacking approach did an excellent implementation using Decision Trees, Random Forest, and XGBoost as base learners with Gradient Boosting as a meta-learner, which managed to record an accuracy of 90%. That indeed puts into great perspective the huge potential it possesses for accuracy measures while predicting in educational setups. The CNN model, which predicted with an accuracy of 89%, showed quite impressive capability in sentiment analysis to acquire further insight into the emotional status of the students. RCNN, Random Forests, and Decision Trees contribute to the possibility of educational data complexity with valuable insight into the complex interrelationships within ML models and educational contexts. The application of the bagging XGBoost algorithm, which attained a high accuracy of 88%, further stamps its utility toward enhancement of academic performance through strong robust techniques of model aggregation. The dataset that was used in this study was sourced from Kaggle, with 1205 entries of 14 attributes concerning adaptability, sentiment, and academic performance; the reliability and richness of the analytical basis are high. The dataset allows rigorous modeling and validation to be done to ensure the findings are considered robust. This study has several implications for education and develops on the key dimensions: teacher effectiveness, educational leadership, and well-being of the students. From the obtained information about student adaptability and sentiment, the developed system helps educators to make modifications in instructional strategy more efficiently for a particular student to enhance effectiveness in teaching. All these aspects could provide critical insights for the educational leadership to devise data-driven strategies that would enhance the overall school-wide academic performance, as well as create a caring learning atmosphere. The integration of sentiment analysis within the structure of education brings an inclusive, responsive attitude toward ensuring students’ well-being and, thus, a caring educational environment. The study is closely aligned with sustainable ICT in education objectives and offers a transformative approach to integrating AI-driven insights with practice in this field. By integrating notorious ML and DL methodologies with educational challenges, the research puts the basis for future innovations and technology in this area. Ultimately, it contributes to sustainable improvement in the educational system.

## Introduction

In an era defined by rapid technological advancements, one of the most promising innovations is the application of machine learning (ML) in various fields [1]. learning, a subset of AI, empowers computers to learn from information and make decisions without being expressly programmed. Its potential to revolutionize industries is evident in sectors like healthcare, finance, and marketing. However, despite the increasing adoption of ML analytics, further research is needed to understand the specific application of the different methods and how they can be used to solve multifaceted problems facing education systems, learning personalisation, performance prediction and adaptive learning environments. Also, most solutions are limited to normal or local scenarios. In addition, few studies delve into global adoptions of ML solutions or the legal requirements that must enforce the implementation of such solutions. These predispositions have not gained sufficient attention in the existing literature, even though they remain critical in cross-cultural and cross-institutional settings, including those that involve international online learning, such as the COIL program, given the different educational and emotional issues likely to emerge.. The amalgamation of machine learning techniques and educational practices holds the promise of reshaping the landscape of learning and pedagogy, offering personalized experiences, refined assessments, and improved outcomes. Machine learning algorithms, driven by patterns and insights extracted from data, are designed to recognise complex relationships and adapt over time. They analyze large datasets to uncover trends, identify correlations, and predict future outcomes. In the context of education, ML algorithms can be trained on enormous student data, encompassing factors such as learning styles, performance history, and engagement metrics. This data-driven approach provides educators and institutions with actionable insights that can be used to tailor instructional methods to individual student needs and preferences. Traditional education systems have often struggled to address the diverse learning profiles of students within a single classroom. This is where machine learning steps in, offering adaptability as a core advantage [2]. Adaptability entails the ability to customize learning experiences to suit each student’s pace and comprehension level. Machine learning algorithms can evaluate a student’s progress and learning trajectory, identifying areas of difficulty and adjusting the curriculum accordingly [3]. This customized approach improves understudy commitment as well as advances a more profound comprehension of the topic.

Sentiment analysis is a potent branch of machine learning entailing the deciphering of human emotions and opinions from written text [4]. Having sentiment analysis integrated within the education system can immensely help to determine what students are doing, engaging in, and understanding about the study matter [5]. By analyzing written assignments, forum posts, and feedback from students, sentiment trends can be analyzed for potential problems, and from there it becomes very easy to intervene with the right support quite early enough to save the situation. This proactive approach contributes to a holistic learning environment that would cater to not only academic growth but also emotional and psychological needs [6]. Another aspect of machine learning, predictive analytics, can have a pivotal role in promoting better academic achievement [7]. Predictive analytics algorithms, through historical data and performance indicators, can predict student outcomes and those who are likely to underperform. An educator can intervene at the early stage, giving extra guidance and resources to the student who needs it. Institutions can also use this information to help develop and give improved tailored curricula, techniques of teaching, and placement of resources [8]. The integration of machine learning into education is an evolutionary process that will revolutionize how both students learn and educators teach. Flexibility in the customization of every student’s learning needs is provided through machine learning, coupled with sentiment analysis to enable a holistic understanding of the state of emotions. Predictive analytics empowers institutions to provide proactive support for students and further fine-tune their educational strategies. It showcases the great potential to create a more inclusive, effective, and student-centred education system where adaptability, sentiment analysis, and academic excellence come into view from the eyes of machine learning [9]. As we delve into the realms of this technological revolution in education, it becomes clear that the journey is only beginning. The cited references shed light on the evolving landscape of education infused with machine learning techniques. By studying these pioneering works, educators, researchers, and policymakers can collectively harness the potential of machine learning to foster a new era of learning that prioritizes personalization, emotional well-being, and academic success. The notation guide of each algorithm and additional abbreviations is shown in “Table 1”.

**Table 1:** Notation guide.

S/N	Notations	Description
1	ML	Machine Learning
2	EDA	Exploratory Data Analysis
3	KNN	k-Nearest Neighbors
4	RF	Random Forest
5	DT	Decision Tree
6	NB	Naïve Bayes
7	XGB	XGBoost
8	NN	Neural Network
9	ANN	Artificial Neural Network
10	SVM	Support Vector Machine
11	EVF	Evimp Functions
12	CART	Classification and Regression
13	PEM	Proposed Ensemble Model

The rest of the paper is ordered as follows: Related Work Section mentions the literature review. The proposed methodology is discussed in the Research Methodology Section. Machine Learning Model Development explains the various ML models details followed by Model Training and Evaluation Section to describe the details of models training and evaluation. Finally, the results and discussion are elaborate in Results and Discussion Sections, respectively.

### Motivation

The motivation behind this study stems from the pressing need to advance educational practices through innovative technologies. As education systems increasingly integrate technology, there is a growing demand for methods that not only enhance academic performance but also address the evolving needs of students and educators. The following key motivations guided this research:

- **Improving Educational Outcomes**: Traditional educational methods often struggle to keep pace with the diverse and dynamic needs of students [59]. By harnessing advanced machine learning and deep learning techniques, this study aims to provide more accurate, data-driven insights that can significantly improve academic outcomes and personalize learning experiences.
- **Enhancing Teacher Effectiveness:** Educators face the challenge of adapting their teaching strategies to cater to the varied needs of students [60]. This research seeks to support teachers by providing tools that enable more precise assessments of student adaptability and performance, thereby enhancing instructional effectiveness and enabling more targeted interventions.
- **Supporting Educational Leadership**: Educational leaders require data-driven strategies to make informed decisions that impact school-wide academic performance and the overall learning environment [61]. This study provides insights that can help leaders implement effective, evidence-based strategies to improve educational outcomes and foster a supportive learning atmosphere.
- **Focusing on Student Well-Being**: Recognizing the importance of student well-being, this research incorporates sentiment analysis to better understand and address students’ emotional and psychological needs. By doing so, it aims to create a more responsive and supportive educational experience that contributes to students’ overall well-being and engagement.
- **Advancing Sustainable ICT in Education**: As the integration of ICT in education grows, there is a need for sustainable and impactful applications of technology [62]. This study aligns with the goals of sustainable ICT by exploring innovative AI-driven approaches that offer long-term benefits for educational improvement and adaptation.

These motivations collectively drive the research, aiming to leverage machine learning and deep learning to transform education into a more effective, personalized, and supportive experience for all stakeholders involved.

## Related work

ML is a powerful tool that has the potential to transform and revolutionize almost all industries, and education is no exception. This paper tries to draw a roadmap of various ways in which machine learning techniques have been used to transform and enhance education [10]. Bringing ML to education has increased its personalization of learning [11], intelligent tutoring systems [12], analysis of educational data [13], and prediction of student performance [14].

In [15], the author presents ten compelling use cases that demonstrate how machine learning can be employed in a practical setting in education. From personalized learning paths and adaptive assessments to systems that recommend intelligent content for students and even systems for early predictions of student performance, examples are demonstrated to illustrate how machine learning can change traditional educational methodologies. With the help of machine learning algorithms, the learning experience can now be individualized to each student’s needs, which is bound to make the experience more engaging and, therefore, the learning performance better. Personalized learning, one of the key concepts in contemporary education, has been enhanced to a great extent by the ML models described by the author in [16]. Learning examples and tendencies from each enable ML models to personalize content and delivery approaches within special care for the learning style of every student, increasing engagement and understanding and leading to improved learning outcomes. Intelligent tutoring systems apply ML to deliver real-time, personalized guidance to help students understand complicated concepts and consolidate weak areas [7]. The data generated through online platforms, assessments, and interactions are huge. The ML techniques analyze this data to obtain insights into student behavior, preferences, and progress. It is this data-driven approach that drives educators to make necessary data-informed adjustments in curriculum and teaching methodologies.

In [14], the author talks about how ML also plays a pivotal role in predicting student performance. By analyzing historical data, demographic factors, and academic indicators, ML models can forecast students at risk of underperforming. Early intervention strategies can then be employed to provide timely support, reducing dropout rates and fostering academic success. Moreover, ML techniques are facilitating the development of smart content recommendation systems [13]. These systems suggest supplementary materials such as articles, videos, and exercises that align with the student’s current topics of study. This encourages self-directed learning and broadens students’ understanding of subjects. The Author in [17], delves into the current state of utilizing machine learning techniques within the context of educational metaverses, and investigates the extent to which machine learning has been integrated into educational metaverse platforms. Educational metaverses encompass virtual and augmented reality environments designed for educational purposes. The authors likely examine the challenges, advancements, and potentials of employing machine learning algorithms to enhance the interactivity, personalization, and overall effectiveness of educational experiences within these immersive virtual environments. However, the integration of ML into education is not without challenges. Issues such as data privacy, algorithmic bias mentioned in [18], and the digital divide must be carefully addressed to ensure equitable and ethical use of ML in education. ML is reshaping the educational landscape. While challenges exist, the benefits of enhanced learning outcomes and tailored educational experiences make the incorporation of ML an essential avenue for the future of education. The idea of versatility has arisen as a critical power driving the change in training, particularly with regard to computerized time. Lately, the worldwide instructive scene has gone through exceptional changes because of the Coronavirus pandemic. Institutes, teachers, and students had to quickly embrace new advancements and educational ways to deal with guarantee continuity in learning [19, 20]. The pandemic highlighted the significance of adaptability in education, with institutions and individuals embracing remote learning platforms such as Zoom and Microsoft Teams as quoted by the author in [21].

This sudden move required adjustments in teaching approaches to make them compatible with the online setting without compromising learning outcomes this view is supported by [22]. Also illustrating the difficulty and triumphs of both educators and learners is [23] and [24]. The point being made was that adaptability was key. Education adaptability It goes beyond the pandemic and includes changes such as modernizing lessons in brick-and-mortar schools to include technology [25]. These investigations focus on important characteristics of technological support for learning. In[63], the authors investigate the effects of technostress on learning environments and performance in order to point out the problems observed among learners. In [64], the authors examine the trend towards development of policy for AI use in higher education, to describe policy requirements for AI regulation. In International online learning, [65] posited self-regulation as a mediator between emotional intelligence and student performance in learning from Latin American universities. Together, these works underscore the need to grasp the psychological, regulating, and technical substrates of the contemporary educational process and, in particular, distance and blended learning.

Adaptive learning systems, for example, as presented in [26], personalize the delivery of content regarding the progress and learning style of individual students. Such systems serve as an example of how adaptability optimizes the learning process itself, based on what every pupil may need for enhanced engagement and comprehension. This concept embraces lifelong learning, evidenced by [27], highlighting the importance of preparing students to fit into an ever-changing job market. This new paradigm requires that educational institutions impart to learners the skills of adaptability—how to acquire new knowledge and competencies during their careers. The transformative potential of adaptability also aligns with competency-based education, as detailed in [28]. This approach emphasizes skill acquisition over traditional course completion, empowering learners to progress at their own pace and demonstrating mastery before advancing Adaptability has emerged as a hallmark of transforming education in the digital age. Having been realized through adaptation to remote learning during COVID, it has shown the need for pedagogical flexibility and technology infusion [19, 20].

Various studies state that adaptability allows us to survive not only in difficult situations but also to improve the quality and effectiveness of education in both traditional and virtual environments. The education evolution will continually be sculpted by the ability of institutions, educators, and learners to embrace change to lend adaptability for better learning outcomes. Sentiment analysis in educational applications is an emerging sub-field of natural language processing that has the potential to help redefine learning. This paper sets out to provide an exhaustive review of how sentiment analysis techniques are applied in the revolution of education toward the capturing and analyzing of emotions, attitudes, and opinions by learners, educators, and stakeholders [29]. Sentiment analysis technology has opened novel avenues to understand the emotional states of learners, as elaborated in references [30, 31]. Gauging students’ reactions towards learning materials is done by sentiment analysis algorithms of written or verbal expressions to be aware of the engagement level and confused topics. This real-time feedback mechanism allows educators to tweak their teaching strategies to better fit the needs of the students, both emotionally and cognitively. Also, from [32] and [22], this sentiment analysis changes methodologies of assessments. In trying to find out how students feel or perceive certain learning engagements, there has always been a loophole in traditional assessments [33]. For instance, sentiment analysis provides non-intrusive ways of understanding students’ feelings towards exams, assignments, and coursework, helping educators design more inclusive and effective evaluation methods. Institutions are using sentiment analysis to enhance student well-being [34]. Works such as [35] and [36] exemplify that sentiment analysis through social media monitoring can be used in detecting students’ emotional struggles, enabling the support units to take action promptly. Such proactivity helps in creating a better learning environment for the students. Sentiment analysis has also been shown to be effective during personalized learning journeys, such as in the cases presented by [37] and [38]. Sentiment analysis through algorithms could assist by suggesting appropriate learning resources for students based on their emotional states and learning preferences, thereby helping learners stay motivated and engaged in their educational journey.

Institutions are using sentiment analysis to enhance student well-being [34]. Works such as [35] and [36] exemplify that sentiment analysis through social media monitoring can be used in detecting students’ emotional struggles, enabling the support units to take action promptly. Such proactivity helps in creating a better learning environment for the students. Sentiment analysis has also been shown to be effective during personalized learning journeys, such as in the cases presented by and [38]. Sentiment analysis through algorithms could assist by suggesting appropriate learning resources for students based on their emotional states and learning preferences, thereby helping learners stay motivated and engaged in their educational journey. Institutions are using sentiment analysis to enhance student well-being [34]. Works such as [35] and [36] exemplify that sentiment analysis through social media monitoring can be used in detecting students’ emotional struggles, enabling the support units to take action promptly. Such proactivity helps in creating a better learning environment for the students. Sentiment analysis has also been shown to be effective during personalized learning journeys, such as in the cases presented by [37] and [38]. Sentiment analysis through algorithms could assist by suggesting appropriate learning resources for students based on their emotional states and learning preferences, thereby helping learners stay motivated and engaged in their educational journey.

However, sentiment analysis in education faces challenges from the contextual sensitivity of linguistic nuances [29]. The validation of truthfulness and fairness of sentiment analysis models, which is discussed in [29] and [32], is important to avoid algorithmic bias and hence ensure fair outcomes. This has therefore brought ML into convergence with a framework of education, which offers adaptive systems on how to infer sentiment analysis for good academic performance. This is an all-encompassing review describing this symbiotic relationship, which demonstrates how on a cumulative basis it reshapes the landscape of education [39]. Machine learning has brought adaptive learning models [40] for personalized education, where learning pathways can be variant and adapted according to the pace, preference, and strength of the learners. It is done through real-time sentiment analysis [41], which models the emotional status of learners [42]. Sentiment analysis, in turn, further boosts adaptability through tracking students’ engagement, confusion, and motivation [43], [44]. By gauging the students’ sentiments, the educators can timely intervene and customize the learning experience for maximum efficacy [45]. This transformative power is in the area of academic excellence as well [46], in which the synergy of adaptability, sentiment analysis, and machine learning sparks pre-emptive measures. Analytic predictive algorithms forecast the performance of students, identifying at-risk underperforming students [47]. Through sentiment analysis, references [48] and [36] demonstrate how affective data inform the design of interventions aimed at improving academic support strategies and thereby increasing student success. As further evidence of this connection between academic success and adaptability, competency-based education has entered the learning environment [49]. Sentiment analysis can be used to detect whether a flexible approach to learning is working for students so that refinements may be made accordingly to achieve optimum success. Further, sentiment analyses will allow institutions to make adjustments in content, pacing, and support systems to achieve higher engagement and attainment [50]. However, there are still some challenges: It thus becomes very important that the sentiment analysis model learns context and nuances [41], as biases may influence the accuracy of emotional assessments [51]. These ethical considerations highlighted in reference [52] point to responsible machine learning integration, mitigating privacy issues and algorithmic biases. Sentiment analysis is, according to Vasilis Bourikas (2023), the most considerable factor in increasing student engagement and building resilience in higher education. It can act as a good tool for identifying those students who are struggling and providing help custom-made to their needs, building good coping methods [53]. In “Machine Learning in Education: How to Boost Efficiency,” Fayrix (2022) deals with the application of machine learning for online education efficiency. The essay goes in-depth about how machine learning can potentially improve personalization in material and the ability for larger and further reach, speed up processes, and lift ROI [54, 55]. Itransition (2022) explores the various implementations of machine learning in education in an article titled “ML in Education: 10 Use Cases, Technologies, Benefits, and Barriers.” The latter discusses ten different applications, among which are personalized learning, new assessment methodologies, and recommendation systems. In “The Impact of Artificial Intelligence on Learning, Teaching, and Education,” the European Commission of 2021 has deeply explored the potential effects of the application of AI on education. The present paper explores how AI can support teaching and learning, also by tackling the challenges on the way toward the full realization of its benefits [56].

Another study focuses on sentiment analysis to further advance student learning [57]. Therefore, the current paper checks for the use of sentiment analysis in identifying underperforming students, providing targeted support, and tracking the academic progress of those students. Another paper [58] underscores the transformational potential of machine learning in education. It emphasizes AI that provides personalized growth opportunities designed to articulate the needs and weaknesses of students individually. In conclusion, it is this combination of machine learning, adaptivity, sentiment analysis, and academic brilliance that makes possible an educational ecosystem pulsating with dynamism. These elements will enrich personal learning journeys, harness emotional insights to predict outcomes, and further better the quality, inclusivity, and efficacy of education using this transformational framework. The future is in harmonizing these elements into a holistic learning environment that takes care of diverse needs and aspirations.

## Research methodology

**Fig 1:**
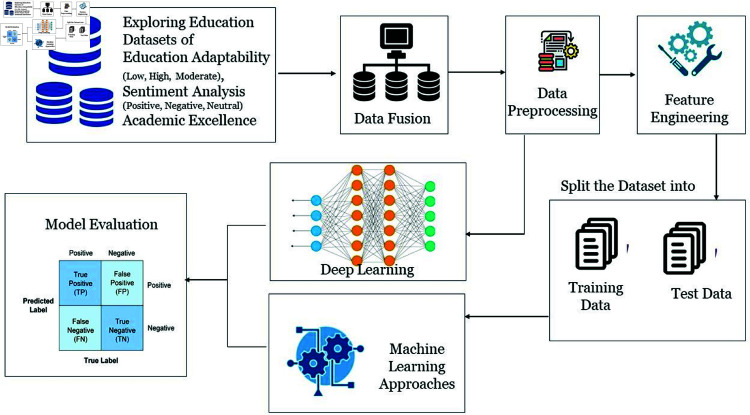
Exploring education through artificial intelligence proposed methodology.

The research method of our study is a step-by-step process that follows the achievement of our research objectives. It starts with the data exploration stage, in which an education dataset that meets the criteria of our research is identified. Subsequently, this research merged various sources of data into an enriched dataset. The preprocessing of data involved data cleansing, handling missing values, and standardization of the format. After performing this preprocessing step, we train Deep Learning classifiers, followed by their assessment of whether or not they have produced predictive accuracy. In parallel, feature engineering was performed to increase the importance of dataset variables. A partitioning resulted in a 75-25 train-test split that allowed the following application of Machine Learning classifiers. The models have been very trained and closely measured for their accuracy and effectiveness. This methodology reflects a comprehensive approach, spanning dataset exploration, aggregation, preprocessing, DL, and ML techniques, culminating in a robust evaluation within the educational context as also shown in Fig 1.

### Data collection and preprocessing

The dataset employed in this study comprises information concerning students’ adaptability, sentiment analysis, and academic excellence. The dataset encompasses a total of 1205 entries, each characterized by 14 attributes, including gender, age, education, organization type, IT pupil status, area, load-shedding, monetary condition, web type, network type, class term, self-learning the executive framework utilization, gadget utilized, and adaptivity level. Before starting examinations, data preprocessing methods were executed to deliver the dataset appropriate for ML algorithms. Missing values were examined, revealing no instances of missing data within the features. Categorical attributes were numerically encoded, while the “Adaptivity Level” feature was transformed into numerical values, with categorical labels “Low,” “Moderate", and “High” replaced by 0, 1, and 2, and for the sentiment analysis, positive and negative sentiments respectively.

**Continuous variables** are those that can take on any value in a specified range and are measured on a scale. In this study, we included the age which is actually a numerical value that can vary continuously. Another continuous variable is the class duration, which measures the length of online classes usually in minutes or hours. Another continuous variable is financial condition, as it represents the condition of the financial status of the student and also can be quantified through an income level or score of financial stability. Self-LMS usage represents another continuous variable because it tracks the number of hours a student uses self-learning management systems every week. There is yet another continuous variable: internet speed, measured in megabits per second (Mbps), which might change from student to student. On the contrary, **categorical variables** are those that represent distinct groups or categories that cannot be measured on a numerical scale. Such variables help to classify the students into various groups for the analysis. For example, gender forms a categorical variable that categorizes the students into different groups such as Male, Female, or Other. Another categorical variable is the education level of the student, indicating whether the student is in school, college, or university. Institution type, on the other hand, distinguishes between public and private institutions of educational institutions. The variable IT student determines whether a student is pursuing an IT-related course of study, categorized as yes or no. Location refers to whether the student lives in an urban or rural area, whereas load shedding reports whether the student suffers from power cuts frequently, which is referred to as Yes or No. The Network type variable, differentiates between internet connections like Mobile Data or Broadband, whereas the Devices used category differentiates the types of devices that the student utilizes for online learning including Smartphones, Laptops, or tablets. Adaptability level is a categorical variable; that is, it classifies the group of students into Low, Medium, and High categories according to their adaptability to online education. With regards to the explicit and explicit description of continuous and categorical variables, the research study lays out the different categories of data to be analyzed and how they contribute to understanding factors that influence the adaptability of the students and their academic achievement.

### Exploratory data analysis (EDA)

**Fig 2:**
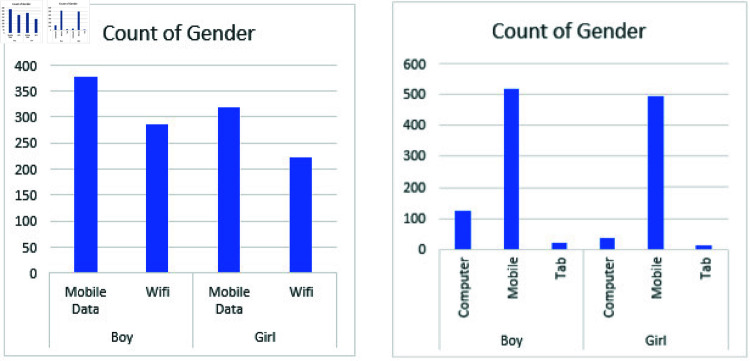
Visualizations comparing gender and Internet types.

Before model development, an exploratory data analysis (EDA) was performed. Descriptive statistics, including means, standard deviations, and quantiles, were computed to glean insights into attribute distributions. Various visualizations, such as bar plots, histograms, and scatter plots, were generated to visualize the characteristics of categorical and continuous variables. As shown in Fig 2(a) and Fig 2(b) most of the students either it’s a boy or a girl prefer the Mobile platform for online education instead of Laptops/computers and they use cellular data. This thing clearly illustrates that they are not attaining the education attentionally. They go through it like an ordinary process, using the cell phone along travel anywhere, would be helpful but the alarming situation is that their number (Students who are using the cellphone for online classes and using cellular data) is increasing rapidly instead of other students who are taking the classes form laptops/computer. Additionally, potential correlations between attributes were investigated through visual exploration.

### Data splitting and standardization

The dataset was split into a training subset and a testing subset in a ratio of 75% for training and 25% for testing. The attributes were separated: input variable, X, from the target variable, “Adaptivity Level,” y. To make the model converge and improve in performance, the input variables were standardized using the StandardAero from the scikit-learn library.

### Correlation matrix

**Fig 3:**
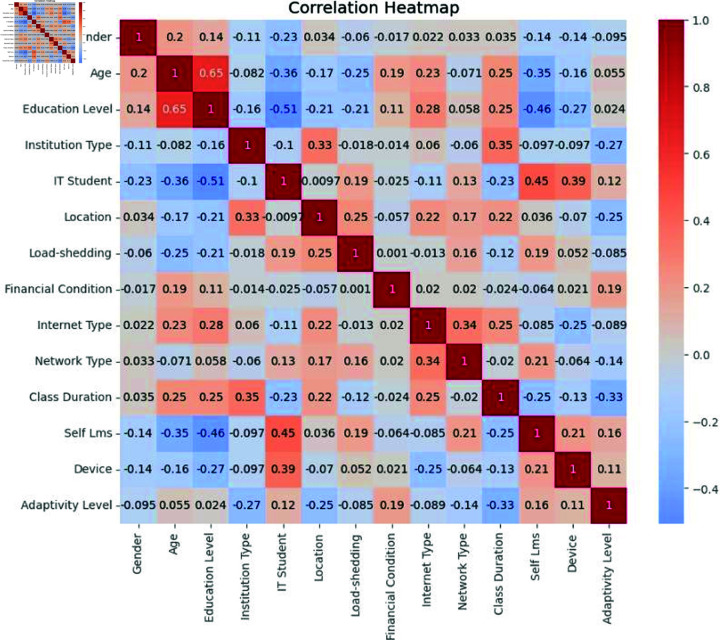
Correlation matrix.

A correlation matrix is a statistical tool that computes the relationship between every variable in a dataset. These coefficients, demonstrate the direction and strength of linear association. It helps in identifying patterns and dependencies between variables, hence helping in feature selection or data exploration. Looking into the correlation matrix will dictate most aspects of data-driven decision-making in finance, scientific study, etc (Fig 3).

## Machine learning model development

Diverse machine learning algorithms were employed for model development:

### Convolutional neural network (CNN)

The present study employed the Convolutional Neural Network (CNN) to identify the influence of image-based attributes on change in education. Built purposely to discard complex spatial components in images, the CNN was suitable for uncovering visual factors associated with adaptability, sentiment analysis, and academic excellence in learners. In general, the basic architecture of the CNN is built upon convolutional layers, pooling layers, and fully connected layers that enable the presented architecture to extract important patterns and relationships within the visual data. The model was trained on the training dataset with an emphasis on minimizing a pre-assigned loss function as displayed in equation (1). Evaluation metrics, including precision and loss, were utilized to survey CNN’s presentation. Fundamental outcomes showed an accuracy of 89%.

$$y={\text{argmax}}_{j}\left(f(x;\theta {)}_{j}+\gamma g(x;\theta )\right)$$ (1)

Where:

The terms are the same as for CNN, with the addition of the g(x;θ) term, which controls the interaction between the CNN and the bounding boxes.

### Recurrent convolutional neural network (RCNN)

Apart from this, the study used a recurrent convolutional neural network to detect temporal dependencies concerning spatial characteristics. In general, RCNN architecture incorporated the power of CNN and recurrent layers to give a complete scenario about students’ adaptability, sentiment analysis, and academic excellence. It captured temporal dependencies along with the spatial characteristics of the sequences of images, considering their spatial characteristics through an RCNN, as shown in equation (2). A model was trained with a particular loss function used to minimize training. The evaluation metrics measured accuracy and loss to identify the RCNN’s performance. The first observation was an accuracy of around 74%.

$$y={\text{argmax}}_{j}\;f(x;\theta {)}_{j}$$ (2)

### XGBoost

For this purpose, the XGBoost algorithm has been adopted. XGBoost is considered one of the best algorithms for the prediction of complex relationships in tabular data because it uses boosting. The sequential construction of decision trees exposes patterns responsible for the adaptability, sentiment analysis, and academic excellence of students as shown in equation (3). Trained over the training data, the XGBoost model has been evaluated using accuracy, precision, recall, F1-score, and others. The calculation shows around 88%.

$$h(x)=f(x)+\sum_{i=1}^{m}\beta_{i}\left[g(x;\theta_{i})+\gamma_{i}h(x)\right]$$ (3)

Where:

The terms are the same as for gradient boosting, with the addition of the i term, which controls the interaction between the weak learners

### Decision tree

The Decision Tree algorithm was applied to all the non-image attributes to make them interpretable. Decision Trees provide clear attribute relations from the straightforward decision paths, which influence the adaptability, sentiment analysis, and academic excellence of students. Equation (5) denotes how: Decision Trees were trained on a training dataset and then evaluated through accuracy metrics to get a clear understanding of their ability to make informed educational predictions based on interpretable rules. Preliminary results show a performance level of about 75%.

$$y={\text{argmax}}_{j}\;p(y=j|x)$$ (4)

### Random forest

Working from the Decision Trees concept, the Random Forest algorithm leveraged the collective intelligence of several trees in its extension. That is to say, it generated several decision trees with slight variations and captured different perspectives on the influences that shape adaptability, sentiment analysis, and excellence in academics for the students. The ensemble nature of the algorithm ensures that the predictions are robust because it mitigates overfitting and accounts for a variety of attribute interactions, as in equation (6). Random Forests were applied to the training and testing datasets, respectively, and an accuracy of about 73% was recorded.

$$y=\frac{1}{n}\sum_{i=1}^{n}h_{i}(x)$$ (5)

### Stacking approach

**Fig 4:**
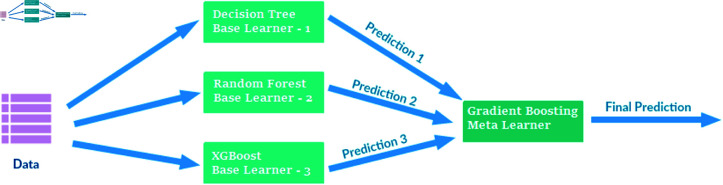
Architecture of the proposed stacked ensemble learning model, illustrating the use of three base learners (decision tree, random forest, and XGBoost) to generate predictions (Prediction 1, Prediction 2, and Prediction 3), which are then combined by a gradient boosting meta-learner to produce the final prediction. This approach leverages the strengths of individual models to enhance overall predictive accuracy.

In analyzing non-image attributes, by applying the stacking hybrid approach DT, RF, XGBoost as a base model and the Gradient Boosting algorithm as a meta-model was employed as shown in Fig 4.

This stacking approach sequentially builds using the base models DT, RF, and XGBoost to enhance predictions, as illustrated in equation (4). This allows an in-depth review of the intricate relationships impacting students’ adaptability, sentiment analysis, and academic performance. The meta-model Gradient Boosting was trained and evaluated on the training dataset using indicative metrics to provide insights into its ability to capture complex attribute interactions. The accuracy of the first results was around 90%.

$$h(x)=f(x)+\sum_{i=1}^{m}\beta_{i}\left[g(x;\theta_{i})\right]$$ (6)

## Analysis

We experienced several algorithms concerning the proposed platform for their accuracy. We want to evaluate thoroughly to find out the most efficient algorithm that gives precise results and reliable outcomes. This would, in turn, help in fine-tuning the performance and reliability of the entire system.

### Analyzing CNN

**Fig 5:**
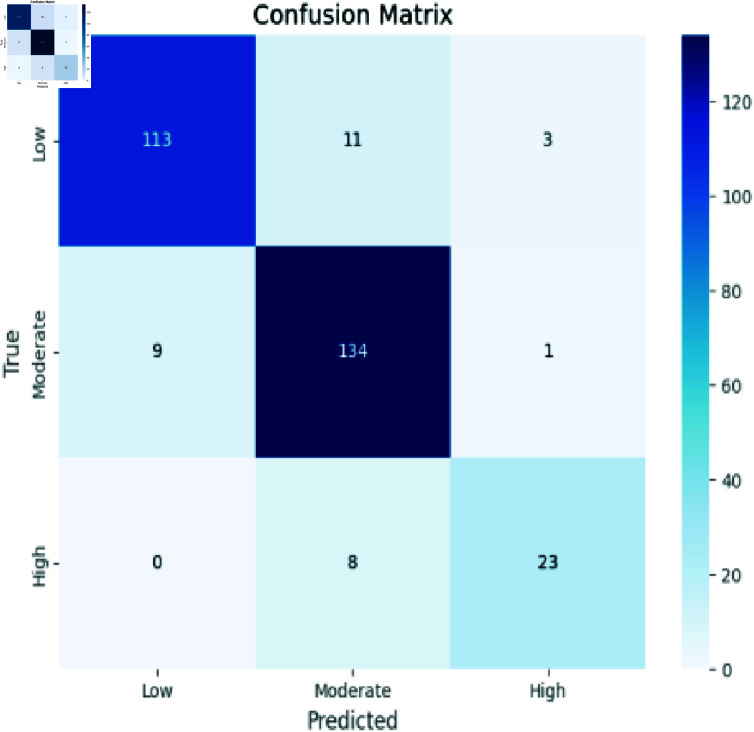
CNN model evaluation.

The confusion matrix in Fig 5 summarizes our model’s performance across Low, Moderate, and High classes. It accurately predicted Low instances (113), Moderate instances (134), and High instances (23). However, misclassifications were observed: predicting Moderate as High (8), Low (11); predicting High as Moderate (1), Low (3); and predicting Low as Moderate (9).

### Analyzing RCNN

**Fig 6:**
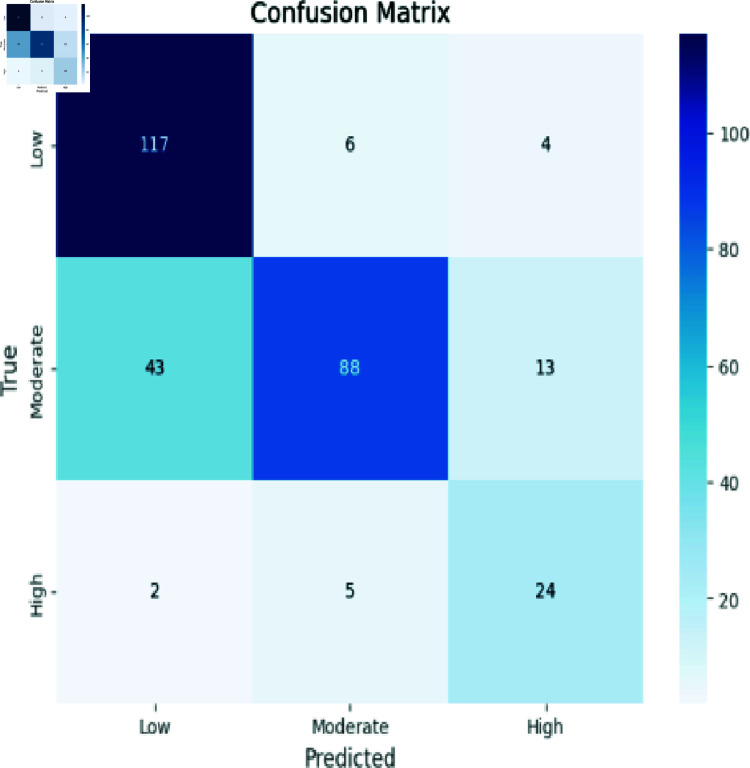
RCNN model evaluation.

Fig 6 demonstrates precision in predicting Low instances (117), Moderate instances (88), and High instances (24). However, some misclassifications emerged: predicting Moderate as Low (66), High as Moderate (5), and High as Low (2). These results provide insight into the model’s.

### Analyzing decision trees

**Fig 7:**
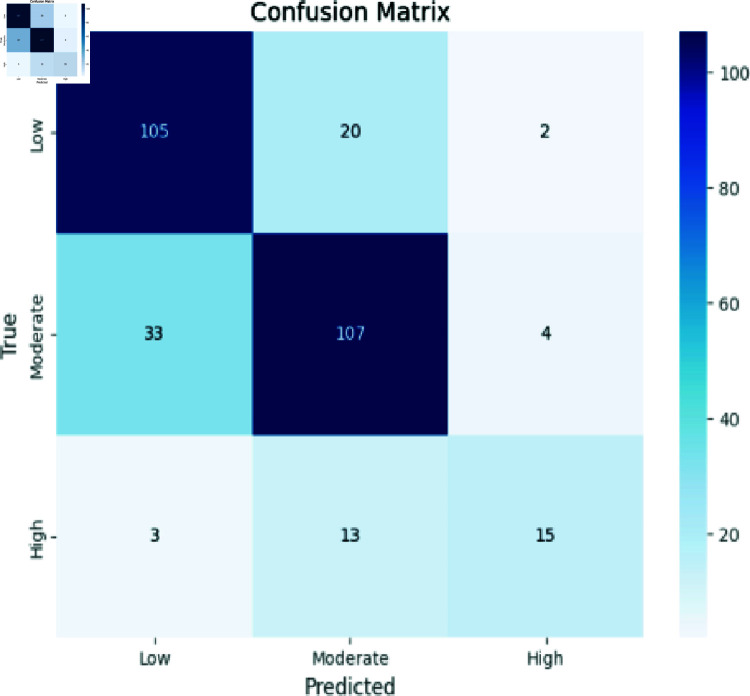
Decision tree evaluation.

Fig 7 demonstrates accuracy in predicting High instances (15), Moderate instances (107), and Low instances (105). However, a degree of misclassification emerged: predicting Moderate as High (13) and Low (20), and predicting High as Moderate (4) and Low (2). These findings accentuate the model’s proficiency within each class, while also revealing areas where misclassifications occurred, particularly between Moderate and High classes, and in the Low class. This matrix serves as a comprehensive snapshot of the model’s performance, illuminating its predictive prowess and areas warranting further scrutiny.

### Analyzing stacking approach

**Fig 8:**
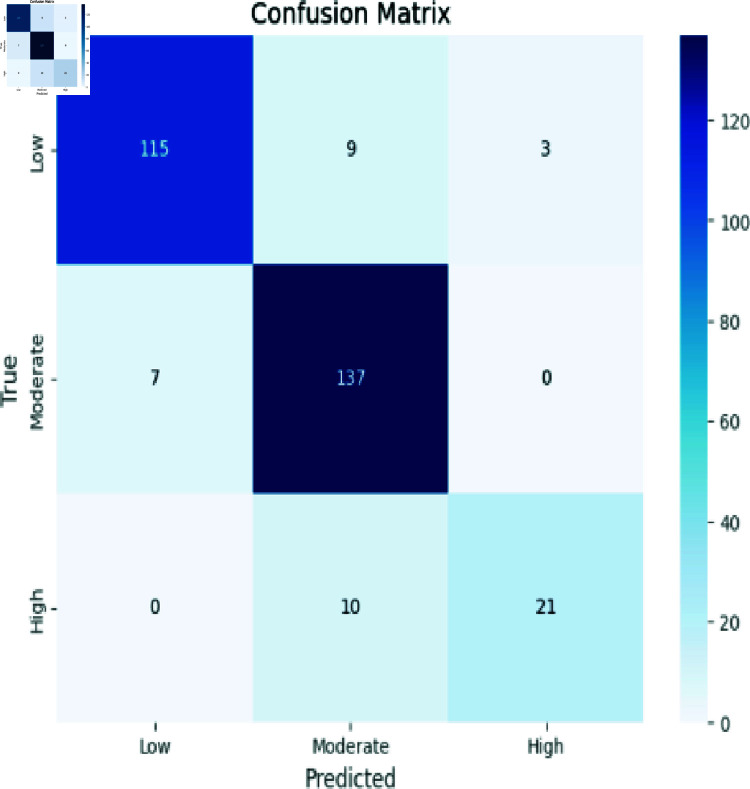
Stacking approach evaluation.

Fig 8 demonstrates Predictions of “Low” were largely aligned with the true class (115 instances), although some instances were misclassified as “Moderate” (7 instances). For “Moderate” predictions, accuracy was evident within the class (137 instances), though misclassifications arose into “High” (10 instances) and “Low” (9 instances). Instances predicted as “High” demonstrated precision within the class (21 instances), while misclassifications were observed as efficacy within each class and shed light on instances where misclassifications occurred, particularly between Moderate and Low and High and Moderate classes “Low” (3 instances). This matrix encapsulates the model’s performance trends across classes, shedding light on its strengths and misclassification patterns.

### Analyzing XG boosting

**Fig 9:**
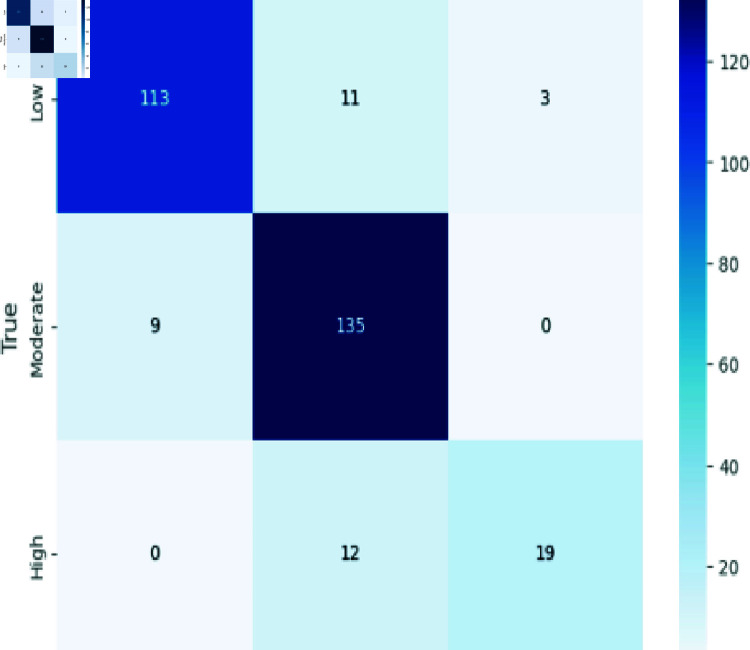
XG boosting evaluation.

Fig 9 Predictions of “Low” displayed alignment with the true class (113 instances), except for instances misclassified as “Moderate” (9 instances). For “Moderate” predictions, concordance within the class (135 instances) was prevalent, alongside misclassifications into “High” (12 instances) and “Low” (11 instances). Instances classified as “High” exhibited precision within the class (19 instances), but misclassifications emerged as “Low” (3 instances). This matrix encapsulates the model’s proficiency within these classes, spotlighting both its accurate predictions and areas warranting further attention.

### Analyzing random forest

**Fig 10:**
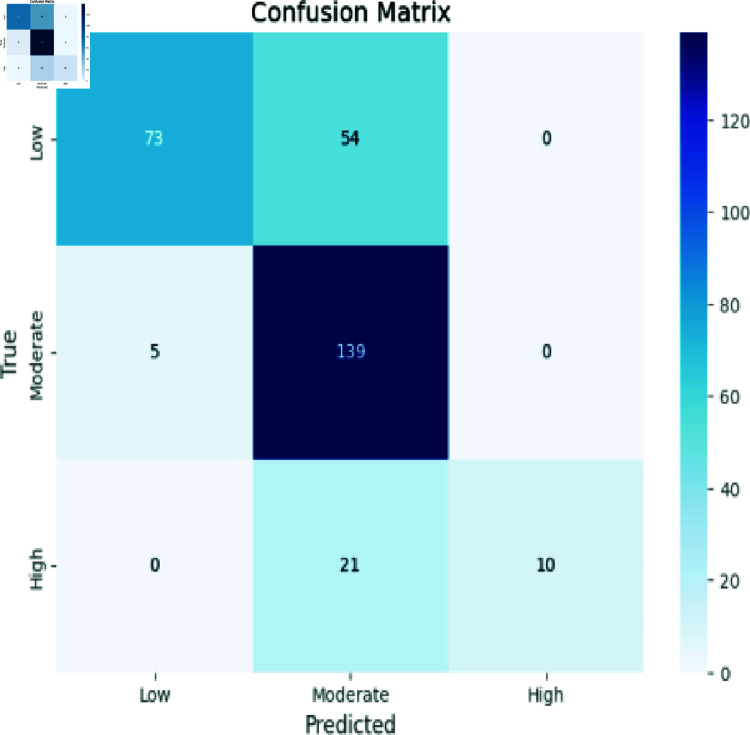
Random forest evaluation.

The confusion matrix in Fig 10 outlines our model’s predictions within the High, Moderate, and Low classes. Instances predicted as “Low” aligned with the true class (73 instances), though misclassifications arose as “Moderate” (5 instances). For “Moderate” predictions, precision within the class (139 instances) was evident, alongside misclassifications as “High” (21 instances) and “Low” (54 instances). Instances classified as “High” displayed precision (10 instances), with no misclassifications, yet this class was not predicted for “Moderate” or “Low.” This matrix encapsulates the model’s effectiveness across classes, emphasizing accurate predictions and areas warranting investigation.

## Image and non-image attributes

For image-based attributes, convolutional neural networks (CNN) and recurrent convolutional neural networks (RCNN) were designed. CNN focused on extracting spatial features from images, while RCNNs considered both spatial and temporal features. Ensemble techniques, including XGBoost, stacking Gradient Boosting meta-model, Decision Tree, and Random Forest, were employed for non-image attributes. These algorithms are renowned for their capability to capture complex relationships within data.

## Model training and evaluation

Models were trained using the training dataset. CNN and RCNN models were trained on image-based attributes, while ensemble models were trained on non-image attributes. The training involved the optimization of respective algorithm-specific objective functions. Model evaluation encompassed diverse metrics appropriate to each algorithm: For CNN and RCNN: Evaluation metrics included accuracy and loss on the testing dataset. For ensemble models: Metrics such as accuracy, precision, recall, and F1-score were employed to gauge model performance.

## Results

In this section, we present the results of the machine learning (ML) and deep learning (DL) classifiers on various assessment parameters, including accuracy, recall, and F-measure. The performance of these classifiers is evaluated based on the precision of AI models in investigating instructional versatility, academic excellence, and sentiment analysis data. Among the classifiers evaluated, the Gradient Boosting Tree (GBT) outperformed others in terms of accuracy.

### Parameters to be evaluated

Precision, accuracy, recall, and F-measure are the key evaluation metrics considered in this study to assess the performance of the ML classifiers, as shown in Table 2. The evaluation involves calculating the specificity (accuracy) and sensitivity (recall) for each classifier to analyze the predicted precision. The accuracy, precision, recall, and F-measure are derived using the following standard formulas:

**Table 2:** Accuracy of ML classifiers.

Classifiers	Accuracy	Precision	Recall	F-Measure
RF	73%	0.94	0.57	0.71
XGBoost	88%	0.93	0.89	0.91
Decision Tree	75%	0.74	0.83	0.78
Gradient Boosting	90%	0.94	0.91	0.92
RCNN	74%	0.72	0.92	0.81
CNN	89%	0.93	0.89	0.91

**Fig 11:**
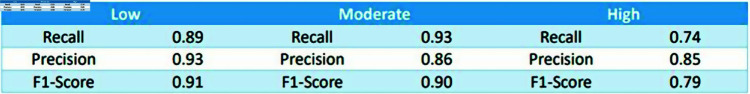
Confusion matrix evaluation of CNN.

**Fig 12:**
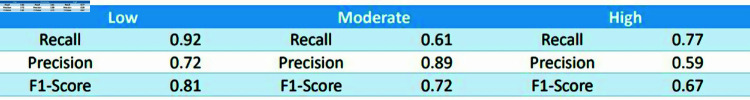
Confusion matrix evaluation of RCNN.

**Fig 13:**
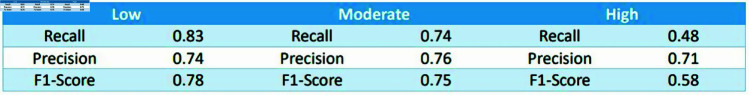
Confusion matrix evaluation of XGB.

**Fig 14:**
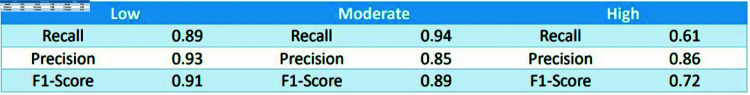
Confusion matrix evaluation of decision trees.

**Fig 15:**
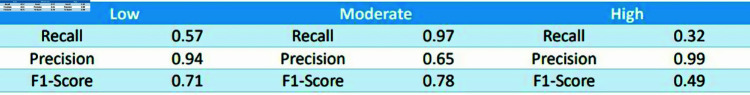
Confusion matrix evaluation of stacking approach.

Accuracy: The ratio of the number of correctly identified instances to the total number of instances in the dataset, as shown in Eq (7). The confusion matrix evaluation scores for CNN, RCNN, XGB, Decision Tree, Stacking approach, and Random Forest are shown in Figs 11-15.

$$\text{Accuracy}=\frac{TP+TN}{TP+FP+TN+FN}\times 100$$ (7)

Precision: The average probability of retrieving relevant information, as shown in Eq (8).

$$\text{Precision}=\frac{TP}{TP+FP}$$ (8)

Recall: The average probability of full retrieval, as shown in Eq (9).

$$\text{Recall}=\frac{TP}{TP+FN}$$ (9)

F-Measure: After calculating the precision and recall, the F-measure combines these two scores. The traditional F-measure is calculated using the following equation:

$$\text{F-Measure}=\frac{2\times \text{Precision}\times \text{Recall}}{\text{Precision}+\text{Recall}}$$ (10)

The recall, precision, and F1-scores of CNN, RCNN, Decision Trees, Gradient Boosting, XGB, and Random Forest are shown in Table 2.

### ROC curves

**Fig 16:**
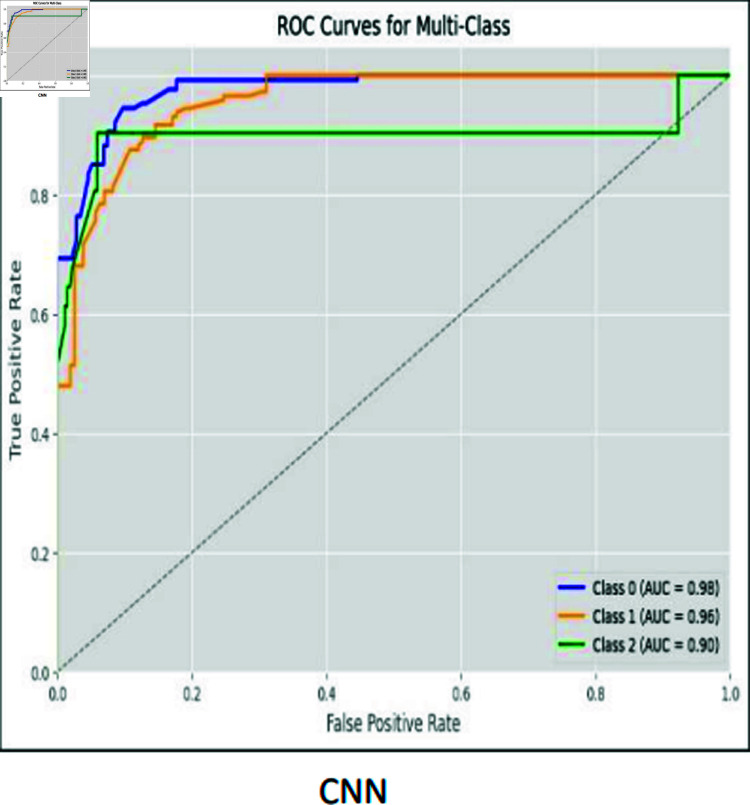
ROC curve CNN.

**Fig 17:**
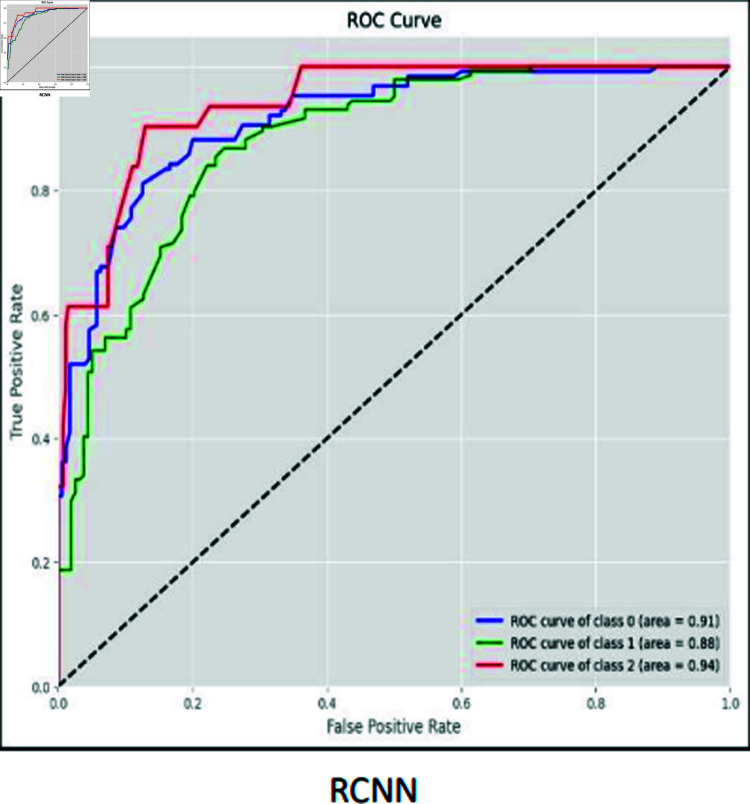
ROC curve RCNN.

**Fig 18:**
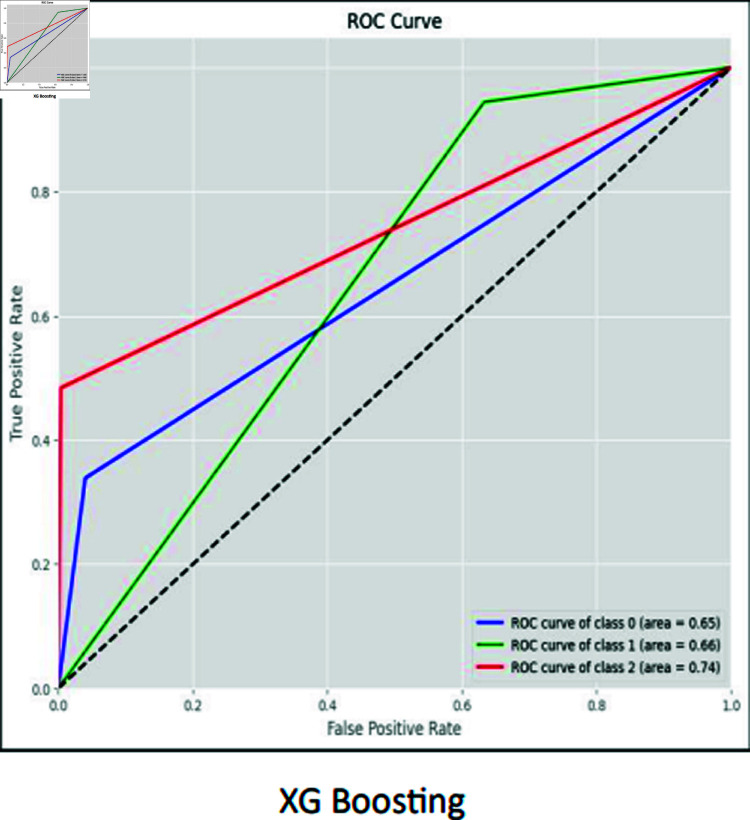
ROC curve stacking meta model gradient boosting.

**Fig 19:**
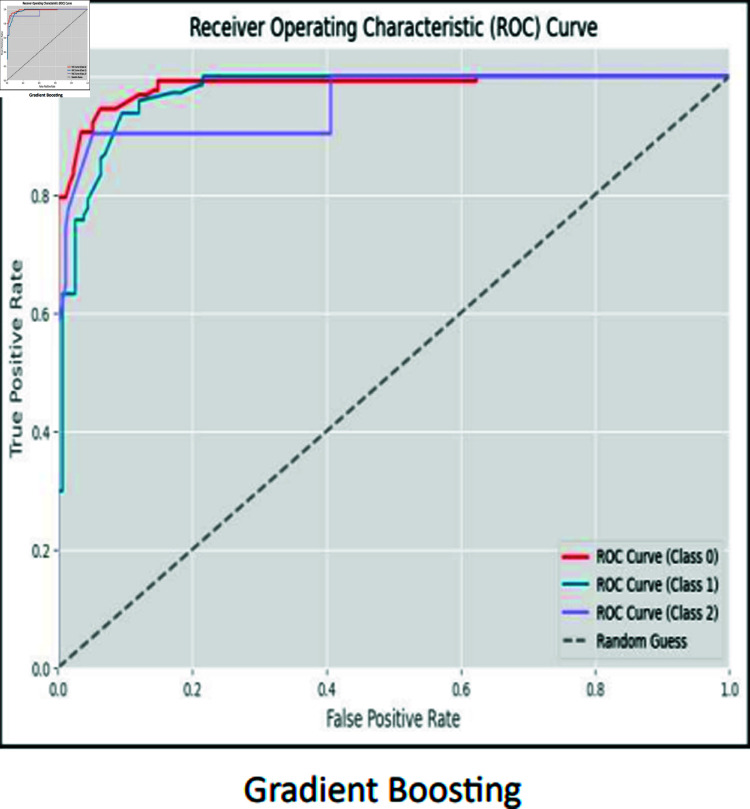
ROC curve XG boosting.

**Fig 20:**
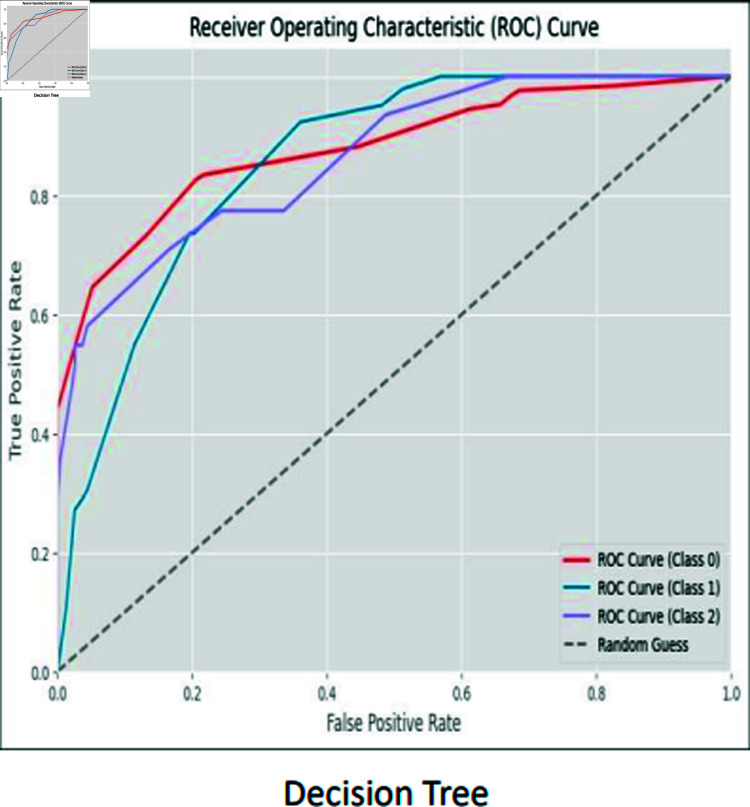
ROC curve decision trees.

**Fig 21:**
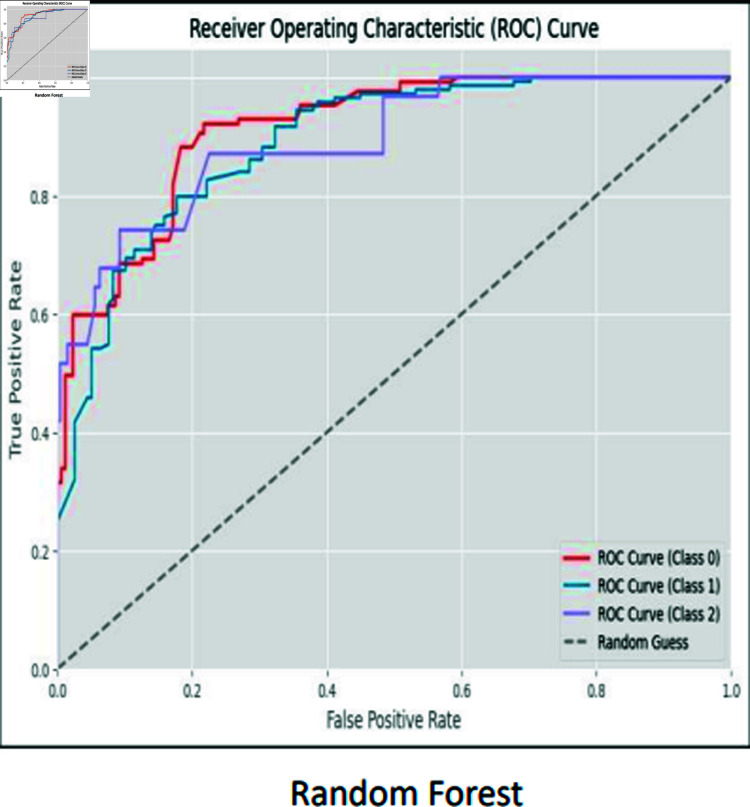
ROC curve random forest.

The ROC curve is used to visually represent the trade-off between the True Positive Rate (sensitivity) and the False Positive Rate (specificity) as the classification threshold varies. The ROC curves for CNN, RCNN, XGB, Decision Tree, Stacking approach, and Random Forest are shown in Figs 16–21.

### ML and DL classifiers accuracy

**Fig 22:**
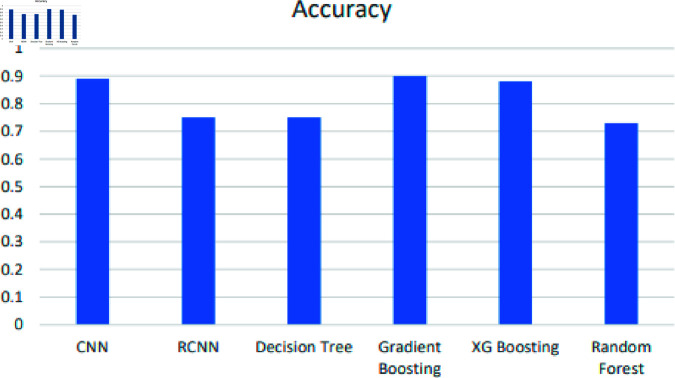
Accuracy chart of ML and DL classifiers.

Table 2 shows the accuracies of various classifiers used in transforming education through machine learning and deep learning techniques for exploring adaptability, sentiment analysis, and academic excellence. The Stacking model achieved the highest performance, while the Random Forest (RF) classifier performed the least. The Gradient Boosting classifier achieved an accuracy of 90%, as shown in Figure 22.

## Discussion

In this section, we provide a thorough interpretation of the results presented in the previous section. The findings indicate that the Gradient Boosting Tree (GBT) classifier, with its 90% accuracy, outperformed other models like Random Forest (RF), which had the least accuracy at 73%. The performance of the classifiers was evaluated using several key metrics, such as accuracy, precision, recall, and F-measure. The stacking model exhibited superior results, showcasing its potential in accurately transforming educational data.

This study’s findings align with previous research in the field of educational technology and machine learning, particularly in the areas of adaptability and sentiment analysis. Recent advancements in deep learning, such as the use of Convolutional Neural Networks (CNN) and Recurrent Convolutional Neural Networks (RCNN), have demonstrated significant promise in extracting relevant patterns from educational data. Our results corroborate these findings, with CNN and RCNN models achieving strong recall and precision values.

Moreover, the ROC curve analysis revealed a strong relationship between the True Positive Rate and False Positive Rate for most classifiers, further supporting the efficacy of the proposed models. As shown in the ROC curves, the Stacking approach with Gradient Boosting demonstrated the most favorable trade-offs between sensitivity and specificity.

The study contributes to the growing body of research on using machine learning and deep learning techniques in educational technology. By leveraging AI models like Gradient Boosting and Stacking, educational institutions can better understand and enhance various aspects of the learning environment, such as adaptability, academic excellence, and sentiment analysis.

We believe that the implications of this study are significant, providing valuable insights for researchers and educators alike. Future research should focus on refining these models and exploring their practical applications in real-world educational settings.

### Theoretical contribution and practical implication

This study presents several significant contributions in the field of educational technology by leveraging the applications of advanced machine learning and deep learning techniques. The key contributions include:

- **Development of a Comprehensive AI Framework**: The research introduces a robust AI framework that integrates a diverse array of algorithms—XGBoost, CNN, RCNN, RF, DT, and a hybrid stacking approach. This framework demonstrates superior performance, with the stacking approach achieving a 90% accuracy, thus providing a highly effective tool for analyzing and improving educational outcomes.
- **Enhanced Sentiment Analysis Capabilities:** By employing CNNs and RCNNs, the study advances sentiment analysis in educational contexts. The CNN approach achieved an accuracy of 89%, showcasing its effectiveness in understanding and interpreting students’ emotional and psychological states. This capability allows for more nuanced insights into student well-being, contributing to a more empathetic and responsive educational environment.
- **Insights into Teacher Effectiveness and Instructional Strategies:** The study’s findings offer valuable insights into how AI can support teachers in refining their instructional methods. The ability to analyze student adaptability and performance data helps educators tailor their teaching strategies to better meet individual student needs, thereby enhancing instructional effectiveness and overall educational quality.
- **Data-Driven Strategies for Educational Leadership:** The research provides educational leaders with actionable insights that can be used to develop and implement data-driven strategies. These strategies are designed to improve school-wide academic outcomes and foster a supportive and efficient learning environment, aligning with contemporary goals for educational leadership.
- **Focus on Student Well-Being**: The incorporation of sentiment analysis into the framework emphasizes the importance of addressing students’ emotional and psychological needs. By offering a more comprehensive understanding of student well-being, the study contributes to creating a more holistic and supportive educational experience.
- **Alignment with Sustainable ICT Goals**: The study aligns with the goals of sustainable ICT in education by providing innovative, AI-driven solutions that promote long-term educational improvement. The application of advanced technologies not only enhances educational outcomes, but also supports the sustainable development of ICT in educational settings.

These contributions collectively advance the application of AI in education, providing a transformative approach that benefits students, educators, and educational institutions alike.

### Limitations

While this study provides valuable insights into the performance of various machine learning and deep learning classifiers in transforming education through adaptability, sentiment analysis, and academic excellence, it is important to acknowledge the following limitations:

- **Data Dependency:** The findings of this study are significantly dependent on the set of datasets employed for the training and testing purposes. Concerning the external validity of the results, we would like to point to the fact that the nature of these datasets may limit the ways in which the system can learn about the educational environment and all its possible variations.
- **Model Interpretability:** Even though other sophisticated deep learning models such as CNN and RCNN presented high overall performance, they are black box types, making it difficult to analyze the results. Different models deal with this aspect differently, and how these models make decisions remains a topic of debate and central to their use in real life.
- **Computational Complexity:** Certain classifiers, including most deep learning models, are computationally intensive and take longer to train. Such a limitation could hamper their viability to solve problems in real-life settings, especially in large-scale education systems in developing nations.
- **Class Imbalance:** Class imbalance in the classifiers’ input dataset may have affected performance. Despite using oversampling and undersampling, more extensive research into enhanced methods to tackle this problem could further improve classifier performance.
- **Scope of Features:** The range of features employed in this work does not exhaust all possible factors affecting the quality of education and educational performance. Future research could extend the involvement of more attributes, including demographic information, institutional resources, and interactions between teachers and students.
- **Limitations in Evaluation Metrics:** Unlike accuracy, precision, recall, and F-measure, other evaluation metrics do not capture all aspects of model performance, especially in scenarios where the dataset is imbalanced. Alternative evaluation methods, such as AUROC or MCC, could potentially provide a better understanding of how well the model truly performs.

## Conclusion and future work

This study makes a substantial contribution to the field of educational technology by applying advanced machine learning and deep learning techniques to transform education. By employing a range of algorithms, including XGBoost, Convolutional Neural Networks (CNN), Region-based Convolutional Neural Networks (RCNN), Random Forest, Decision Trees, and a hybrid stacking approach (integrating Decision Tree, Random Forest, and XGBoost as base models with Gradient Boosting as the meta-model), the research achieved a notable accuracy of 90% with the stacking method. The CNN approach, demonstrating an accuracy of 89%, proved effective in sentiment analysis, while the RCNN, Random Forest, and Decision Trees provided valuable insights into the complex interactions between machine learning and educational contexts. The bagging XGBoost algorithm, with an accuracy of 88%, underscored its potential for enhancing academic performance. Utilizing a robust dataset from Kaggle, which includes 1205 entries and 14 attributes related to adaptability, sentiment, and academic excellence, this study has achieved significant outcomes. The developed system enhances teacher effectiveness by enabling educators to tailor teaching strategies to individual student needs, thereby improving instructional effectiveness. Educational leaders can leverage these insights to implement data-driven strategies that enhance school-wide academic outcomes and create a more supportive learning environment. Moreover, the focus on student well-being through sentiment analysis contributes to a more holistic and responsive educational experience. This research aligns with the goals of sustainable ICT in education, providing a transformative approach to educational improvement through AI-driven insights.
